# Explainable machine learning for predicting postoperative length of stay after gastrectomy: a nationwide study using XGBoost and SHAP

**DOI:** 10.3389/fmedt.2025.1732580

**Published:** 2025-12-05

**Authors:** Tsunehiko Maruyama, Kazuto Ikezawa, Hideo Suzuki, Tomohiro Kurokawa, Yoshimasa Akashi, Tatsuya Oda

**Affiliations:** 1 Department of Surgery, Mito Saiseikai General Hospital, Mito, Japan; 2 Department of Gastroenterological Surgery, University of Tsukuba, Tsukuba, Japan; 3 Saiseikai Research Institute of Health Care and Welfare, Tokyo, Japan; 4 Department of Gastroenterology, Tsukuba Memorial Hospital, Tsukuba, Japan; 5 Research and Development Center for Precision Medicine, University of Tsukuba, Tsukuba, Japan; 6 Department of Medical Epigenomics Research, Fukushima Medical University, Fukushima, Japan

**Keywords:** artificial intelligence, DPC data, gastric cancer, explainable AI, length of stay

## Abstract

**Background:**

Gastric cancer remains a major cause of cancer-related morbidity and mortality. Despite advances in surgical and perioperative care, prolonged hospitalization continues to strain healthcare systems. Predicting postoperative length of stay (LOS) could support personalized care and efficient resource allocation. Japan's nationwide Diagnosis Procedure Combination (DPC) database provides real-world data for large-scale analysis, but no study has applied machine learning to predict LOS after gastrectomy.

**Methods:**

This retrospective study included 26,097 patients who underwent gastrectomy between 2017 and 2022 at 472 hospitals in Japan. Using XGBoost, we developed a predictive model based on 1,433 admission-time variables extracted from the DPC database. Model performance was evaluated using Root Mean Squared Error (RMSE) and Mean Absolute Error (MAE) in a five-fold cross-validation. SHAP values were used to interpret feature importance.

**Results:**

The final model achieved an RMSE of 3.74 and MAE of 2.82 days. Key predictors of LOS included surgical procedure (laparoscopic distal gastrectomy and open total gastrectomy), designated cancer hospital, hospital size, peritoneal dissemination, and admission ADL score. SHAP analysis revealed that Laparoscopic distal gastrectomy and higher hospital volume were associated with shorter LOS, while open total gastrectomy was associated with longer LOS.

**Conclusions:**

We developed a machine learning model that predicts postoperative length of stay with an error range of 2–4 days using admission data. This proof-of-concept study demonstrates the feasibility of predicting length of stay from admission data, showing that explainable AI can replicate intuitive patterns in surgical oncology while simultaneously identifying unexpected insights from administrative data. These findings highlight the clinical potential of explainable AI for perioperative workflow optimization.

## 1 Introduction

According to GLOBOCAN statistics, gastric cancer ranks fifth in both incidence and mortality worldwide (1). Advances in perioperative management have led to a reduction in postoperative complication rates and shorter hospital stays following gastric cancer surgery. Curative gastrectomy remains the only potentially curative treatment for patients with resectable gastric cancer. However, despite improvements in surgical techniques and perioperative care, this procedure is still associated with a high rate of complications (30%–44%) and a mortality rate of 3%–4% (2, 3). In recent years, the aging population, increase in comorbidities, and diversity of surgical risk have contributed to a consistent incidence of postoperative complications, prolonged hospital stays, increased healthcare costs, and greater burdens on healthcare providers.

The Diagnosis Procedure Combination (DPC) system, introduced in Japan in 2003, is the largest medical data in Japan, collecting data from approximately 1,700 acute care hospitals nationwide (4). It has been widely used in various clinical studies.

Machine learning, a subfield of artificial intelligence (AI), has evolved to flexibly capture complex nonlinear relationships, particularly when handling large-scale datasets, and is becoming increasingly common in medical research (5). The combination of AI with big data is highly synergistic, and analyses based on both are expected to play a key role in improving healthcare systems. Accurate prediction of postoperative length of stay (LOS) could help reduce the burden on healthcare staff and improve patient outcomes. Although several studies have analyzed short-term outcomes after surgery using Japan's DPC data (6), none have applied machine learning to these data. Therefore, the objective of this study was to identify factors associated with LOS after gastrectomy for gastric cancer using machine learning applied to the DPC database, and to develop a predictive model capable of estimating postoperative LOS at the time of admission. We emphasize the proof-of-concept nature of this work and discuss the methodological, clinical, and healthcare-system implications.

## 2 Methods

This study was conducted in accordance with the Transparent Reporting of a multivariable prediction model for Individual Prognosis Or Diagnosis (TRIPOD) guidelines (7).

### 2.1 Source of data

**Figure 1:**
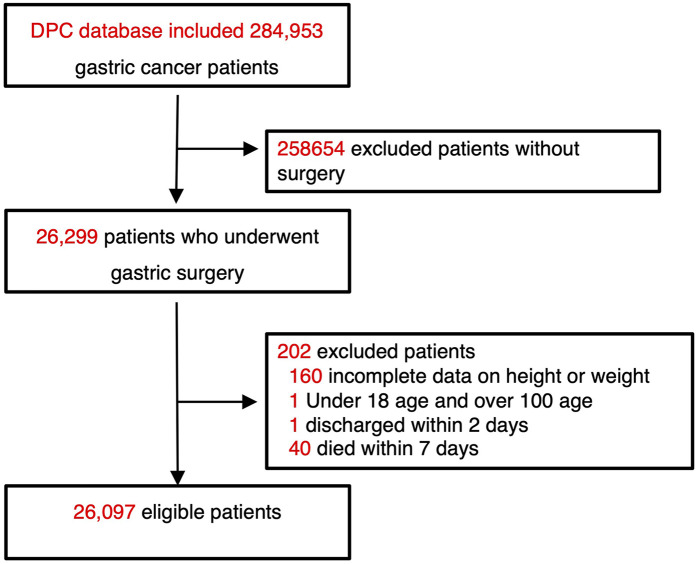
Flowchart of patient selection from the DPC database. Among 284,953 gastric cancer patients, 26,097 who underwent surgery and met inclusion criteria were analyzed.

We obtained anonymized DPC data from Medical Data Vision Co., Ltd. Patients were identified using the International Classification of Diseases, 10th Revision (ICD-10) code C16 (malignant neoplasm of the stomach). A total of 284,953 gastric cancer patients hospitalized at 472 institutions between August 1, 2017, and July 31, 2022, were initially identified. Among them, 26,299 surgical cases were extracted using the Japanese surgical classification code, known as the K-code. The exclusion criteria were as follows: (1) unknown height or weight; (2) age under 18 or over 100 years; (3) hospital stay shorter than 2 days; and (4) death within 7 days of admission. After applying these criteria, 26,097 patients were included in the final analysis (Figure 1).

In Japan, healthcare staff are required to select appropriate DPC codes when admitting patients (8). The DPC database includes a wide range of clinical information such as patient background characteristics, comorbidities, admission and discharge details, diagnoses, surgeries, and medications. To prepare the dataset for analysis, all statistical information related to each hospitalization was reviewed. The original DPC dataset consisted of multiple CSV files containing mixed data types, including numerical variables (e.g., age, Barthel Index), categorical variables (e.g., surgical codes, hospital type), and free-text diagnosis fields converted to ICD-10 codes automatically standardized by the DPC system. All available data were thoroughly examined, including variable contents, formats, patterns, missing values, and invalid entries, to ensure standardization. The data files were then merged for analysis, cleaned, and filtered. Columns or rows with unreadable content due to numerical or format errors were removed. Missing values were encoded as “NA” and processed according to XGBoost's built-in handling of missingness. To ensure dataset consistency prior to model training, all variables were converted to standardized numerical values using one-hot encoding. A total of 1,433 variables were extracted as features for analysis.

### 2.2 Data analysis

To build the machine learning model and identify factors with high predictive importance, we employed eXtreme Gradient Boosting (XGBoost), a representative ensemble learning method. XGBoost is a tree-based machine learning algorithm widely used for classification and regression tasks, and is known for its high performance and computational efficiency as an advanced implementation of gradient boosting (an ensemble learning method) (9). The analytical procedure using XGBoost was as follows. We used the Python XGBoost library to calculate feature importance and constructed a predictive model for postoperative LOS in gastric cancer patients as the target variable. The model's predictive accuracy was evaluated using Root Mean Squared Error (RMSE) and Mean Absolute Error (MAE). Five-fold cross-validation was performed by splitting the data into five subsets, using one subset for testing and the remaining four for training. The evaluation metric was the average of performance across all five folds (folds 0–4). XGBoost was selected because it generally outperforms random forests when handling large tabular datasets containing mixed data types, missing values, and complex nonlinear interactions. Furthermore, gradient boosting decision tree models have repeatedly demonstrated high predictive performance for clinical outcomes in tabular medical datasets, often surpassing traditional methods and other machine learning models such as random forests (10, 11).

To interpret the model's predictions, we used SHapley Additive exPlanations (SHAP), a model-agnostic method that helps explain the outputs of machine learning models (12). SHAP is a model-agnostic interpretability method widely applicable across various machine learning models. SHAP is based on cooperative game theory, where each feature is treated as a “player” contributing to the prediction. A feature's SHAP value represents its average marginal contribution across all possible combinations of features. Formally, SHAP calculates this value by evaluating the difference in model output when the feature is included vs. excluded. This allows for a mathematically consistent measurement of feature contribution within complex models like XGBoost (13, 14). It enables the quantification and interpretation of the contribution of each explanatory variable to the model's predictions, even in complex models with multiple features. A positive SHAP value indicates that the variable contributes to a longer postoperative LOS, while a negative SHAP value indicates that the variable contributes to a shorter LOS.

The data supporting the findings of this study are available from the University of Tsukuba. However, due to licensing agreements, the data are not publicly accessible. Data may be obtained from the corresponding author upon reasonable request and with permission from the University of Tsukuba.

This study was conducted in accordance with the Declaration of Helsinki and was approved by the Ethics Committee for Saiseikai Research Institute of Health Care and Welfare (Approval No. R03-01-03). Because this study was based on secondary analysis of administrative data, individual patients could not be identified. Informed consent was waived due to the anonymized nature of the dataset. Clinical trial number: not applicable.

## 3 Results

### 3.1 Patient characteristics and surgical outcomes

The clinicopathological characteristics of the patients are summarized in Table 1. Of the 26,097 patients, 17,864 (68.5%) were male and 8,223 (31.5%) were female. The median age was 72 years (range, 19–98 years), and the median body mass index (BMI) was 22.4 kg/m^2^ (range, 11.1–42.3). The median total hospital stay was 13 days (range, 2–290 days), and the median postoperative LOS was 11 days (range, 0–30 days). A total of 114 patients (0.4%) died within 28 days after surgery.

**Table 1:** Baseline characteristics of patients undergoing gastrectomy for gastric cancer.

Factor	Value	*N* = 26,097	%
		Number of cases	
Gender, *n* (%)	Male	17,864	68.5
	Female	8,233	31.5
Age (years)	median (range)	72.0 (19.0–98.0)	
Height (cm)	median (range)	162.0 (105.0–193.0)	
Weight (kg)	median (range)	58.2 (30.1–129.5)	
BMI	median (range)	22.4 (11.1–42.3)	
Length of hospital stay (day)	median (range)	13.0 (2.0–290.0)	
Length of hospital stay after surgery (day)	median (range)	11.0 (0.0–30.0)	
UICC T, *n* (%)	0	25	<0.1
	is	30	0.1
	1	10,648	40.8
	2	3,851	15.2
	3	5,043	20
	4	4,659	18.4
	X	1,018	4
UICC N, *n* (%)	0	15,890	62.9
	1	3,768	14.9
	2	2,613	10.3
	3	1,906	7.5
	4	2	<0.1
	X	1,099	4.3
UICC M, *n* (%)	0	22,994	91
	1	1,177	4.7
	X	1,109	4.4
Number of hospital beds, *n* (%)	199≥	916	3.5
	200≤, 499≥	13,660	52.3
	500≤	11,521	44.1
Designated cancer hospitals, *n* (%)	+	21,222	81.3
	-	4,875	18.7
Death within 28 days after surgery, *n* (%)	+	114	0.4
	-	25,984	99.6

Regarding the type of surgery, laparoscopic distal gastrectomy was the most common procedure, performed in 11,509 patients (44.1%), followed by open distal gastrectomy in 6,946 patients (26.6%) (Table 2).

**Table 2:** Distribution of surgical procedures in the study cohort.

Surgical codes in Japan	Surgical procedure	*N* = 26,097	%
		Number of cases	
K6552	Open distal gastrectomy	6,946	26.6
K655-22	Laparoscopic distal gastrectomy	11,509	44.1
K655-42	Open proximal gastrectomy	417	1.6
K655-52	Laparoscopic proximal gastrectomy	987	3.9
K6572	Open total gastrectomy	4,436	17.0
K657-22	Laparoscopic total gastrectomy	1,802	6.9

### 3.2 Feature importance and model performance (RMSE and MAE)

**Figure 2:**
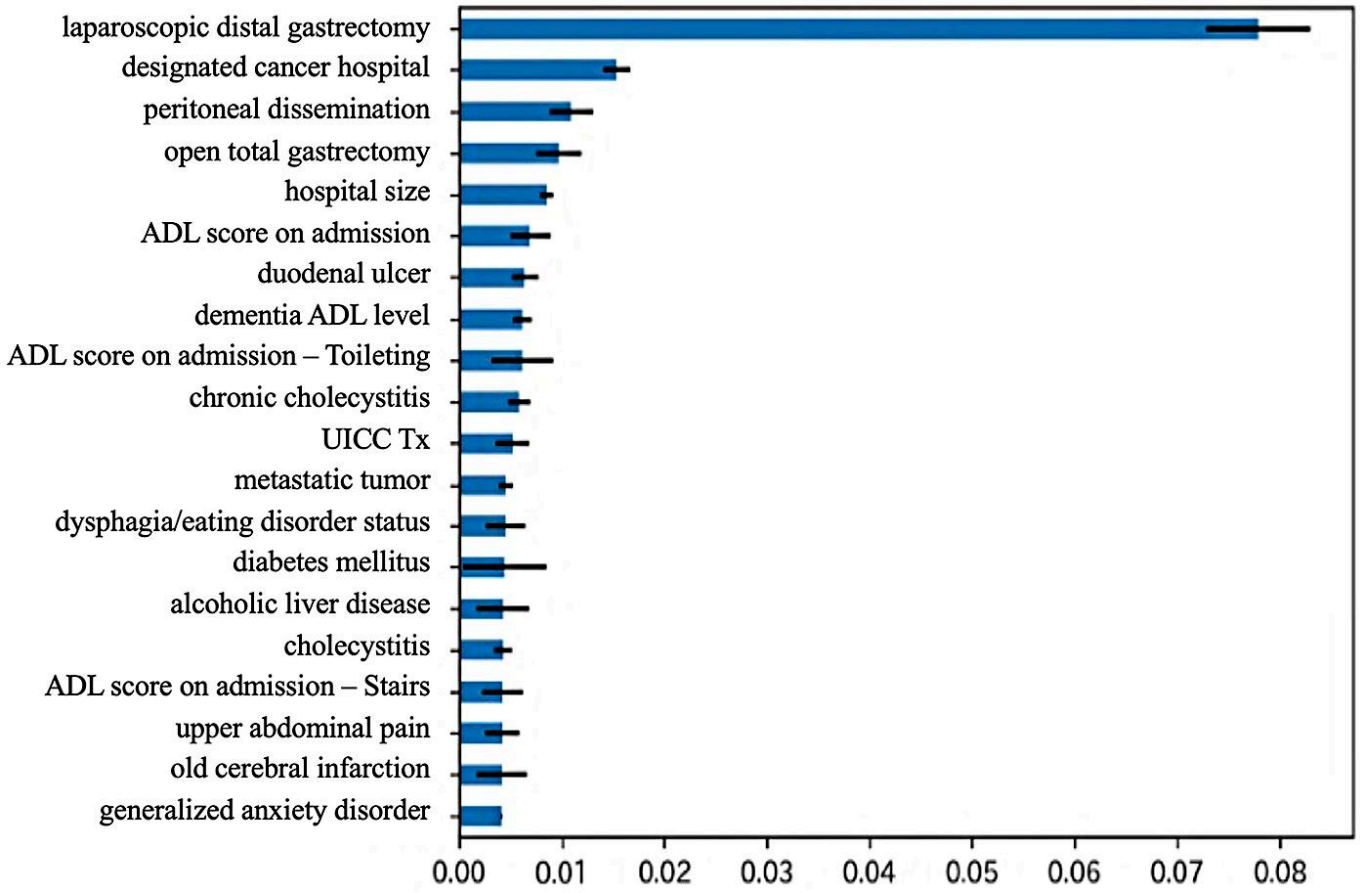
Top features contributing to postoperative length of stay predicted by XGBoost. Features such as laparoscopic distal gastrectomy, designation as a cancer hospital, presence of peritoneal dissemination, open total gastrectomy, hospital size, and the ADL (Activities of Daily Living) score at admission had the greatest impact on predicted length of stay.

The relative importance of each predictive feature was assessed using the feature importance metrics provided by the XGBoost model. The most important predictors included laparoscopic distal gastrectomy, designation cancer hospital, presence of peritoneal metastasis, open total gastrectomy, hospital size, and the Activities of Daily Living (ADL) score at admission (Figure 2). The model achieved a RMSE of 3.74 (standard error 0.03) and a MAE of 2.82 (standard error 0.01), indicating that postoperative LOS could be predicted with an error of approximately 2–4 days (Table 3).

**Table 3:** Performance metrics of the machine learning model for predicting postoperative length of stay.

Metric	Mean	SD
RMSE	3.74	0.03
MAE	2.82	0.01

### 3.3 SHAP-based interpretation of predictive features

**Figure 3:**
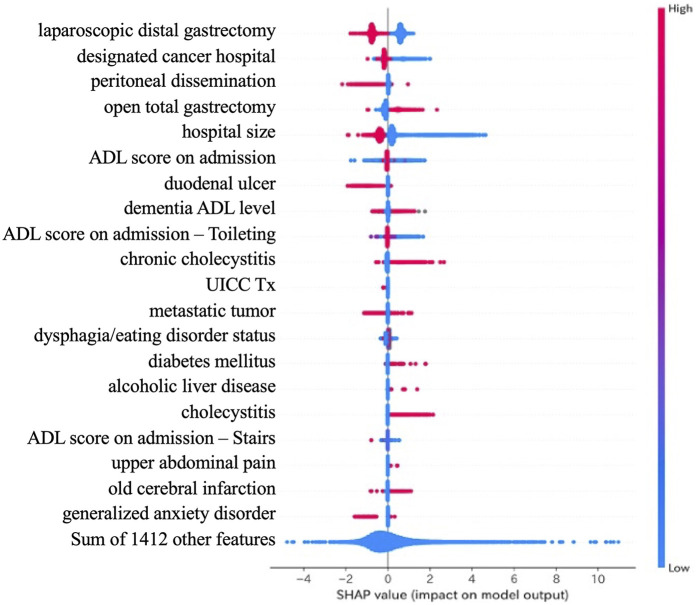
SHAP summary plot for feature interpretability. SHAP (SHapley Additive exPlanations) values visualize the influence of each feature on the model's output across all patients. Each dot represents an individual patient. Red dots indicate higher feature values, while blue dots indicate lower values. Features are ranked by overall impact. Laparoscopic surgery and large hospital size were associated with shorter length of stay, while open total gastrectomy contributed to prolonged hospitalization.

Figure 3 shows the SHAP comprehensive plot generated from the model. In this plot, explanatory variables contributing most to the model's prediction are ranked from top to bottom. Each row corresponds to a variable, and each dot represents a single patient. Red dots indicate higher values of the corresponding feature, while blue dots indicate lower values. The horizontal axis shows the SHAP value, which corresponds to the log-odds in logistic regression. Red dots on the left side suggest that higher feature values are associated with shorter postoperative LOS, whereas blue dots on the left indicate that lower values are associated with longer LOS.

Laparoscopic distal gastrectomy was associated with shorter postoperative LOS, while open total gastrectomy was associated with longer stays. Undergoing surgery at designation cancer hospital, or larger hospitals size to shorten LOS. Interestingly, the presence of peritoneal metastasis also showed a trend toward shorter LOS, while the impact of ADL score at admission was inconclusive.

## 4 Discussion

Accurately predicting LOS is essential for hospital bed management and staffing decisions (15). However, LOS is influenced by numerous factors, making it difficult to forecast with high precision. machine learning enables computers to uncover patterns in data without explicitly programmed rules (16). Given the data-driven nature of machine learning, which does not require assumptions, we hypothesized that it could help predict LOS in hospitalized patients. Using Japan's DPC database and machine learning techniques, we developed a model capable of predicting postoperative LOS after gastric cancer surgery with an error margin of approximately 2–4 days based on RMSE and MAE.

In recent years, an increasing number of studies have developed clinical prediction models using machine learning. Pera et al. developed a 90-day postoperative mortality prediction model for gastric cancer using a development cohort of 3,182 cases and a validation cohort of 266 cases, achieving an Area Under the Curve (AUC) of 0.844. They identified six major contributing variables: age, hospital size, preoperative albumin and hemoglobin levels, type of gastrectomy, and a history of chronic obstructive pulmonary disease (17). Similarly, Zhang et al. analyzed 1,481 cases of thoracoscopic surgery for lung cancer and developed a model to predict postoperative LOS, reporting an AUC of 0.72–0.80. Key predictors included operation time, age, and serum creatinine (18). Additionally, Lu et al. predicted postoperative complications (19), while Jo et al. and Cho et al. predicted length of stay using XGBoost across the broad surgical dataset (20, 21). However, no studies exist that utilize nationwide administrative data specifically focused on gastrectomy. Shi et al. applied SHAP interpretation to gastrointestinal surgery, but their study targeted a small, single-institution prospective cohort (22).

In our model for predicting postoperative LOS in gastric cancer, key contributing factors included the type of gastrectomy, designation cancer hospital, hospital size, and the patient's ADL score. Laparoscopic distal gastrectomy emerged as the most influential factor in shortening LOS, consistent with previous analyses using Japan's National Clinical Database (NCD) (23). Conversely, open total gastrectomy was associated with longer LOS, which is a clinically intuitive finding for surgeons. While the impact of hospital size on postoperative outcomes has been previously debated (24, 25), our results suggest that hospital size plays a more significant role than patient-related factors in predicting LOS. Similarly, surgeries performed at designation cancer hospital in Japan were associated with shorter LOS. The presence of peritoneal metastasis was identified as a high-impact variable, and SHAP analysis further indicated that it contributed to a shorter postoperative LOS. This finding diverges from typical clinical expectations, highlighting a potential discrepancy between clinical intuition and machine learning–based predictions. The finding that peritoneal dissemination was associated with shorter postoperative LOS contradicts typical clinical expectations. One possible explanation is that patients with peritoneal metastasis often undergo diagnostic or palliative rather than curative surgery, leading to earlier discharge or transfer to other facilities postoperatively. Such inconsistencies underscore an important challenge for the future application of machine learning in clinical practice. Importantly, these relationships are correlations based on data, not causal relationships. Since machine learning identifies statistical patterns without prior assumptions, observed relationships should not be interpreted as direct causation.

This study has several limitations. Unlike the prospective randomized dataset analyzed by Shi et al. (22), our study utilized retrospective administrative data. While our cohort is significantly larger and multi-center, prospective data may offer higher internal validity. It is a retrospective analysis using a claims-based administrative database. This study is based on secondary use of Japan's DPC administrative claims data, which lacks detailed clinical information such as tumor staging, postoperative complication classification, and preoperative laboratory values. Additionally, variability in coding practices and differences in data entry accuracy across institutions may have influenced the performance of the machine learning model. Nonetheless, the XGBoost algorithm is robust to such noise and missing data. During training, the algorithm learns the optimal split direction for missing values, allowing it to handle incomplete data without requiring imputation or feature removal (26). Furthermore, the study period overlaps with the COVID-19 pandemic, which influenced discharge policies. External or temporal validation is needed to assess generalizability.

A major strength of this study is that, to our knowledge, it is the first to develop a machine learning-based model using Japan's DPC data to predict postoperative LOS following gastric cancer surgery. Furthermore, the model achieved a high degree of accuracy, with an error margin of only 2–4 days. Being able to predict LOS at the time of admission and identify the relative importance and directionality (positive or negative) of contributing factors may facilitate effective perioperative interventions. Despite limitations, the model has potential clinical applications. First, it may aid in bed management by providing early LOS estimates. Second, it may enable earlier multidisciplinary interventions for patients predicted to have prolonged stays. Third, it may support communication with patients and families by offering realistic LOS expectations. These applications require prospective validation and careful integration into clinical workflows.

Future research should include external validation on other datasets, temporal validation across different time periods, and adaptation to international healthcare systems. Integrating additional clinical information, including tumor stage and postoperative complications, will further enhance predictive performance and clinical utility.

## 5 Conclusions

We developed and interpreted an XGBoost-based model to predict LOS after gastric cancer surgery using Japanese DPC data. This model demonstrated reasonable accuracy and identified both intuitive and non-intuitive predictors. This proof-of-concept study highlighted the feasibility of predictions based on administrative data, while emphasizing the need for external validation, and demonstrating the potential of explainable AI for perioperative decision support in oncology.

## Data Availability

The raw data supporting the conclusions of this article will be made available by the authors, without undue reservation.
